# Diabetes Mellitus and Heart Failure With Preserved Ejection Fraction: Role of Obesity

**DOI:** 10.3389/fphys.2021.785879

**Published:** 2022-02-15

**Authors:** Aneesh Dhore-patil, Tariq Thannoun, Rohan Samson, Thierry H. Le Jemtel

**Affiliations:** ^1^Section of Cardiology, Department of Medicine, Tulane University School of Medicine, New Orleans, LA, United States; ^2^Tulane University Heart and Vascular Institute, New Orleans, LA, United States

**Keywords:** obesity, heart failure with preserved ejection fraction, diabetes mellitus, weight loss surgery, visceral adipose tissue, epicardial adipose tissue

## Abstract

Heart failure with preserved ejection fraction is a growing epidemic and accounts for half of all patients with heart failure. Increasing prevalence, morbidity, and clinical inertia have spurred a rethinking of the pathophysiology of heart failure with preserved ejection fraction. Unlike heart failure with reduced ejection fraction, heart failure with preserved ejection fraction has distinct clinical phenotypes. The obese-diabetic phenotype is the most often encountered phenotype in clinical practice and shares the greatest burden of morbidity and mortality. Left ventricular remodeling plays a major role in its pathophysiology. Understanding the interplay of obesity, diabetes mellitus, and inflammation in the pathophysiology of left ventricular remodeling may help in the discovery of new therapeutic targets to improve clinical outcomes in heart failure with preserved ejection fraction. Anti-diabetic agents like glucagon-like-peptide 1 analogs and sodium-glucose co-transporter 2 are promising therapeutic modalities for the obese-diabetic phenotype of heart failure with preserved ejection fraction and aggressive weight loss *via* lifestyle or bariatric surgery is still key to reverse adverse left ventricular remodeling. This review focuses on the obese-diabetic phenotype of heart failure with preserved ejection fraction highlighting the interaction between obesity, diabetes, and coronary microvascular dysfunction in the development and progression of left ventricular remodeling. Recent therapeutic advances are reviewed.

## Introduction

Heart failure with preserved ejection fraction (HFpEF) is a growing epidemic (Owan and Redfield, 2005). Unlike heart failure with reduced ejection fraction (HFrEF), myocardial contractility is near normal in HFpEF, and impaired left ventricular (LV) relaxation/increased stiffness leads to pulmonary congestion and thereby dyspnea, pulmonary hypertension, and exercise intolerance (Becher et al., 2013; Andersson and Vasan, 2014; Borlaug, 2014). Currently, HFpEF is the leading cause of hospitalizations in patients > 65 years. It will overcome HFrEF as the leading cause of heart failure (HF) within the next 10 years (Lam et al., 2011; Liu et al., 2013). The increasing prevalence of HFpEF and lack of guideline-directed therapy, has rekindled interest in its pathophysiology (Borlaug, 2020; Mishra and Kass, 2021).

The cornerstone of HF is LV remodeling. In HFrEF, systolic dysfunction leads to eccentric hypertrophy with LV wall thinning and replacement fibrosis. In HFpEF, the LV wall thickens leading to concentric hypertrophy (LVH) (Heinzel et al., 2015) with impaired myocardial relaxation/increased stiffness leading to LV diastolic dysfunction (LVDD) (LeWinter and Meyer, 2013) and ultimately HFpEF. Age (Cheng et al., 2009), hypertension (Verdecchia et al., 1995), obesity (Woodiwiss et al., 2008), diabetes (T2D) (Eguchi et al., 2005), and renal dysfunction (Pluta et al., 2015) contribute to LV concentric remodeling. Distinct clinical HFpEF phenotypes are increasingly being recognized (Samson et al., 2016). Phenotyping HFpEF allows tailoring therapeutic modalities for concentric LV remodeling reversal and eventually, better outcomes. The obese-diabetic phenotype of HFpEF is extremely common (Samson et al., 2016) and associated with poor outcomes (Yusuf et al., 2003).

Obesity is the main driver of T2D with 90–95% of patients with T2D being obese (Mozaffarian et al., 2015). Obesity and T2D overlap in the development and progression of HFpEF (Altara et al., 2017). In the present review, we reviewed articles related to HFpEF and T2D. We conducted a literature search using PubMed, Embase, Ovid, and Cochrane databases and searched terms like “HF,” “T2D,” “HFpEF,” “Obesity,” “LVDD,” “epicardial adipose tissue (EAT),” and “visceral adipose tissue (VAT).” Arranged by hierarchy we reviewed randomized clinical trials, followed by registries and then cohort studies. This review first addresses how obesity affects LV remodeling and fosters low-grade systemic inflammation/microvascular dysfunction and thereby HFpEF (McHugh et al., 2019; Piche et al., 2020). Specific contributions of T2D to inflammation (Tsalamandris et al., 2019), coronary microvascular dysfunction (CMD) (Di Carli et al., 2003), and cardiac myocytes diastolic Ca^2+^ handling (Eisner et al., 2020) are then reviewed. Last, we address the clinical implications of obesity and T2D on HFpEF outcomes before reviewing emerging therapeutic options.

## Effects of Obesity on the Heart

### Obesity and Left Ventricular Concentric Remodeling

The obesity-LV concentric remodeling association was first reported in observational studies and later confirmed in several community-based cohorts (Peterson et al., 2004b; Wong et al., 2004; Powell et al., 2006; Avelar et al., 2007; Woodiwiss et al., 2008; Turkbey et al., 2010; Gidding et al., 2013; Kishi et al., 2014; Reis et al., 2014; Bello et al., 2016; Fliotsos et al., 2018; Razavi et al., 2020; Yan et al., 2020). The correlation between weight loss and decrease in LV mass and not between weight loss and decline in blood pressure (BP) after metabolic surgery is further evidence of the central role of obesity in the pathogenesis of LV concentric remodeling (Jhaveri et al., 2009; Rider et al., 2009; Owan et al., 2011; Kurnicka et al., 2018). However, the loose correlation between obesity-induced LV concentric remodeling and LVDD suggests that obesity may impair LV diastolic function through other mechanisms than obesity heightened cardiac pre-and afterload (Russo et al., 2011). Obesity-induced increase in myocardial triglycerides (TGs) content and myocardial energetics impairment may worsen LVDD (Peterson et al., 2004a; Rider et al., 2013; Piche and Poirier, 2018; Rayner et al., 2018).

Not unexpectedly, obesity is now a recognized risk factor for incident HFpEF (Packer and Kitzman, 2018; Pandey et al., 2018; Savji et al., 2018). Incident HFpEF correlates more closely with visceral adipose tissue (VAT) mass than body mass index (BMI) (Neeland et al., 2013; Cordola Hsu et al., 2021). Peak aerobic capacity is inversely and independently related to intra-abdominal fat, abdominal adiposity is a strong risk factor for all-cause mortality, and CT measured VAT predicts incident hospitalization in patients with HFpEF (Tsujimoto and Kajio, 2017; Haykowsky et al., 2018; Rao et al., 2018). In the Irbesartan in heart failure with preserved ejection fraction (I-PRESERVE) trial (Massie et al., 2008) 71% of the patients had a BMI > 26.5 kg/m^2^ and 55% of the patients in the Phosphodiesterase-5 inhibition to improve clinical status and exercise capacity in HFpEF (RELAX) trial had a BMI > 35 kg/m^2^ (Haass et al., 2011; Reddy et al., 2019). Women had a relatively greater waist circumference (an indirect measure of VAT) than men in the prospective comparison of angiotensin receptor -neprilysin inhibitor with ARB global outcomes in HFpEF (PARAGON-HF) trial (McMurray et al., 2020). A table regarding the salient features of important trials in HFpEF has been listed in Table 1.

**TABLE 1:** Major heart failure with preserved ejection fraction trials with role of obesity in outcomes.

Major heart failure with preserved ejection fraction trials					
**Name**	**Study type**	**N**	**N (BMI > 30 kg/m^2^)**	**Treatment modality**	**Main outcomes**
I-PRESERVE (Haass et al., 2011)	RCT	4,128	1,409 (34%)	Irbesartan	• Irbesartan did not improve outcomes • BMI >35 kg/m^2^ associated with worse CV outcomes (HR 1.27, *p* 0.011)
PARAGON-HF (McMurray et al., 2020)	RCT	4,796	2,357 (49.1%)	Sacubitril-Valsartan	Sacubitril-Valsartan did not improve outcomes • No subgroup analysis in obese population (HR 0.87, *P* 0.06)
RELAX (Reddy et al., 2019)	RCT	216	81 (38%)	Sildenafil	• Sildenafil did not improve quality of life or exercise capacity • BMI >35 kg/m^2^ associated with greater systemic inflammation, worse exercise capacity and worse quality of life
TOPCAT (Huynh et al., 2019)	RCT	1,751	1,135 (64.8%)	Spironolactone	• In patients from the Americas with obesity (BMI >30 kg/m^2^) spironolactone did improve outcomes (HR 0.62 *p* 0.001)
EMPEROR PRESERVED (Anker et al., 2021)	RCT	2,997	1,343 (45%)	Empagliflozin	• Empagliflozin improved composite of CV death or HF hospitalization (HR 0.73 *p* < 0.001) • Did not improve all cause death Not as effective in BMI > 30 KG/m^2^ (HR 0.85 *p* > 0.05)

*HR, Hazard ratio; RCT, Randomized clinical trial.*

### Obesity and Sodium Retention

Obesity leads to HFpEF by increasing renal tubular sodium reabsorption and plasma volume expansion (Bickel et al., 2001; Kotsis et al., 2010; Obokata et al., 2017). The overproduction of aldosterone in obesity occurs through 2 pathways: 1- renin-angiotensin system activation stimulates aldosterone secretion from the adrenal cortex and the adipocytes (Faulkner et al., 2018). 2-leptin directly stimulates adrenal cortical cells (Faulkner et al., 2018). Natriuretic peptides reduce aldosterone levels but in obesity, there is increased neprilysin activity that curtails their impact on reducing aldosterone secretion (Wang et al., 2004).

Hyperaldosteronism also stimulates the accumulation and inflammation of EAT leading to increased loco-regional and systemic inflammation (Iacobellis et al., 2016; Packer, 2018b).

### Obesity and Low-Grade Systemic Inflammation

White adipose tissue (AT) accumulates in multiple depots. The subcutaneous depot accounts for around 80% of the total AT (Chait and den Hartigh, 2020). Visceral and other ectopic AT depots (EAT, perivascular, hepatic pancreas renal, and skeletal muscle) accounts for the remaining 20% (Chait and den Hartigh, 2020). Visceral AT refers to the intra-abdominal accumulation of mesenteric and omental AT that can be measured by single-slice CT at the level of L4–L5 or the umbilicus and by multiple slice imaging by MRI (Le Jemtel et al., 2018).

Weight gain leads to AT accumulation through adipocyte hypertrophy or hyperplasia. While expanding VAT becomes dysfunctional and inflamed thereby promoting low-grade systemic inflammation (Lumeng et al., 2007). Increasing BMI correlates with a circulating level of inflammatory markers like C-reactive protein (CRP), interleukin (IL) -6, P selectin, vascular cell adhesion molecule 1, plasminogen activator inhibitor 1, and tumor necrotic factor-alpha (TNF-α) (Osborn and Olefsky, 2012; McNelis and Olefsky, 2014). However, circulating inflammatory markers do not reliably reflect the degree of VAT and systemic inflammation (Le Jemtel et al., 2018).

After undergoing hypertrophy, VAT shifts from an anti-inflammatory state that facilitates AT angiogenesis and lipid storage to a pro-inflammatory state with production of monocyte chemoattractant protein- (MCP1), C-X-C motif chemokine 12 leukotriene B4, and colony-stimulating factor 1 that promote proliferation of classically activated macrophages and macrophages AT infiltration (McLaughlin et al., 2017; Reilly and Saltiel, 2017).

**FIGURE 1:**
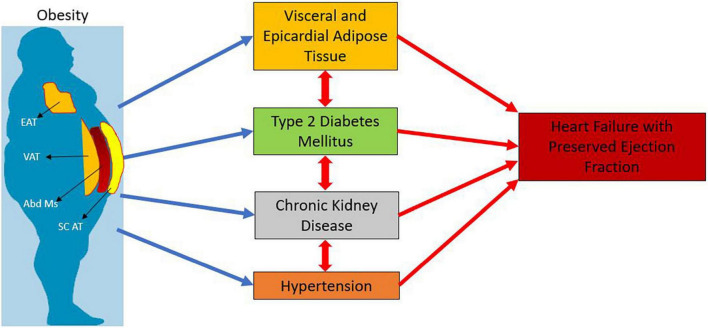
Interplay of obesity and type 2 diabetes with heart failure with preserved ejection fraction. EAT: Epicardial Adipose Tissue; VAT: Visceral Adipose Tissue; Abd Ms : Abdominal Muscles; SC AT : Subcutaneous Adipose Tissue.

Adipogenesis modulates the AT remodeling process and hypoxia is the trigger behind angiogenesis, extracellular matrix remodeling, and inflammation (Crewe et al., 2017; Vishvanath and Gupta, 2019). Inflammatory VAT mediates the production of reactive oxygen species (ROS) and low nitric oxide (NO) that induce mitochondrial dysfunction and activate Nod-like receptor protein 3 (NLRP3) inflammasome (Abad-Jimenez et al., 2020) (Figure 1).

#### Low-Grade Inflammation and Microvascular Dysfunction

Low-grade systemic inflammation worsens cardiovascular diseases (Dhorepatil et al., 2019; Ghoneim et al., 2020a,b). It triggers/heightens an endothelial inflammatory response in the coronary microvasculature (Paulus and Tschope, 2013). In turn, inflammation of the coronary microvascular endothelium alters cardiomyocyte elasticity/function and increases myocardial deposition of collagen that impairs myocardial relaxation and enhances myocardial fibrosis resulting in LVDD and HFpEF (Franssen et al., 2016). Endothelial adhesion molecules enable the infiltration of inflammatory cells that generate hydrogen peroxide (H_2_O2). High oxidative stress uncouples NO synthase (eNOS), reduces NO availability, and decreases soluble guanylate cyclase (sGC) stimulation that lowers the activity of cyclic guanosine monophosphate (cGMP) and protein kinase G (PKG). Low PKG activity leads to cardiomyocytes hypertrophy and decreases titin phosphorylation that increases LV stiffness (Franssen et al., 2016).

Microvascular inflammation is associated with increased production of inducible NOS (iNOS that reduces the protein unfolded response (Paulus, 2020). Suppression of the unfolded protein response may lead to interstitial accumulation of destabilized protein (Paulus, 2020). Microvascular inflammation with macrophages and secretion of transforming growth factor-beta (TGF) results in LV deposition of high tensile collagen (Paulus, 2020).

Lastly, microvascular rarefaction and Sirtuin 3 (SIRT 3) dependent defect in the endothelial cell metabolic programing and angiogenesis may affect the progression of perivascular and myocardial fibrosis in HFpEF (Zeng and Chen, 2019) (Figure 1).

#### Adipocyte Dysfunction and Heart Failure With Preserved Ejection Fraction

The role of adipocyte dysfunction in the development of obese-HFpEF is still evolving. Adipocyte homeostasis is maintained by the modulation of pro-inflammatory and anti-inflammatory cytokines. Obesity leads to an excess of pro-inflammatory cytokines and adipokine dysregulation. Adipose tissue exerts an endocrine effect via adipokines. Adipocyte dysfunction caused by obesity leads to alteration in adipokine levels that promotes LV remodeling and ultimately, HfpEF (Berezin et al., 2020). Elevated leptin levels in obesity are associated with cardiac/renal fibrosis (Packer, 2018a) and increased aldosterone production and sodium retention.

Low adiponectin levels in obesity contribute to an increase in the risk for cardiovascular (CV) disease (Shibata et al., 2009). Adiponectin levels are reduced HFpEF and elevated in HFrEF. *In vitro* adiponectin has multiple beneficial effects such as stimulation of AMP-activated protein kinase (AMPK)-dependent and extracellular-signal-regulated kinase (Barouch et al., 2003) signaling in cardiac myocytes and endothelial cells. Adiponectin reduces LVH and fibrosis, activates endothelial nitric oxide synthase system to and increases NO production (Kobayashi et al., 2004; Ouchi et al., 2004). These beneficial effects have led to an increasing interest in adiponectin as a therapeutic target (Achari and Jain, 2017).

Resistin is an adipocytokine secreted in macrophages by pro-inflammatory cytokines (Lau et al., 2017). Increased resistin levels promote microvascular inflammation, endothelial dysfunction, and vascular smooth muscle proliferation (Acquarone et al., 2019). In elderly patients without HF, serum levels of resistin predict incident HFpEF and HFrEF (Butler et al., 2009). Resistin levels are elevated in patients with HF, but it does not independently predict an adverse outcome (Brankovic et al., 2018). The roles of visfatin, omentin, and other adipocytokines are less well established and an area of active research (Berezin et al., 2020).

### Visceral Adipose Tissue and Heart Failure With Preserved Ejection Fraction

Accumulation of VAT when obesity worsens plays a major role in the development and progression of cardiometabolic conditions. In T2D, VAT is a strong predictor of insulin resistance (Lebovitz and Banerji, 2005) and increased cardiometabolic risk (Rawshani et al., 2020). The inability of the body to cope with unrestricted energy intake leads to VAT expansion that mediates most of the detrimental impact of obesity on clinical outcomes.

In the Multi-Ethnic Study of Atherosclerosis (MESA) (Rao et al., 2018), patients with increased VAT had an independently increased risk of incident HFpEF hospitalization (HR 2.24; 95% C.I. 1.44–3.49). Subcutaneous AT (Sc AT) was not associated with HFpEF. Both VAT and EAT were associated with incident HFpEF hospitalization in the Jackson Heart Study population of African Americans (Rao et al., 2021). Epicardial AT was the only significant variable which predicted all-cause mortality and there was a trend toward increased all-cause mortality seen in VAT (Rao et al., 2021). There was no significant trend seen with S c. AT (Rao et al., 2021) (Table 2). These findings point toward the additive effects of VAT and EAT in the obese-HFpEF phenotype.

**TABLE 2:** Relationship of visceral and epicardial adipose tissue with incident heart failure with preserved ejection fraction.

Name	Study design	N	M	F	Incident HFpEF			Key findings
					N	HR	95% C.I.	
MESA_*EAT*_ (Kenchaiah et al., 2021)	Prospective Cohort Study	6,785	47%	53%	167	1.42	1.25–1.62	• EAT associ ated with increased risk of HFpEF not HFrEF • Elevated EAT conferred a greater risk of HF in women when compared to men
MESA_*VAT*_ (Rao et al., 2018)	Prospective Cohort Study	1,806	48.4%	51.6%	34	2.24	1.44–3.49	• VAT associated with incident HFpEF but not HFrEF • No gender-specific differences in HFpEF incidence
Jackson heart study_*EAT*_ (Rao et al., 2021)	Prospective Cohort Study	1,386	34%	66%	77	1.15	1.08–1.22	• In African American patients, EAT and VAT are independently associated with incident HFpEF
Jackson heart study_*VAT*_ (Rao et al., 2021)	Prospective Cohort Study	2,844	35%	65%	168	1.12	1.06–1.18	• Increased EAT is independently associated with all-cause mortality even after adjusting for comorbidities
								• Increased VAT is also associated with all-cause mortality, but the association is not significant after adjusting for comorbidities
								•SC AT is not associated with incident HFpEF or all-cause mortality

*EAT, Epicardial Adipose tissue; VAT, Visceral Adipose tissue; HFpEF, Heart failure with preserved ejection fraction; HFrEF, Heart failure with reduced ejection fraction; SC AT, Subcutaneous Adipose tissue.*

In patients with obese-HfpEF, VAT accumulation is associated with LVDD and positively correlates with increased LV mass (Abbasi et al., 2015), sphericity, and lower end-diastolic volumes (Neeland et al., 2013). Effects of VAT are also gender specific, with women at baseline tending to have higher VAT% and in HFpEF having worse hemodynamics (Sorimachi et al., 2021). Women with increased VAT and HFpEF have higher exercise-induced LV filling pressures compared with their counterparts with lesser VAT (Sorimachi et al., 2021).

### Pericardial/Epicardial Adipose Tissue and Heart Failure With Preserved Ejection Fraction

Increased pericardial/EAT is independently associated with both obesity and T2D (Yafei et al., 2019). EAT is twice as metabolically active as normal white AT and is involved a great degree of lipolysis and free fatty acid release (FFA) (Marchington et al., 1989). Excess circulating FFA levels lead to increased cardiac TG deposition. As EAT directly lies on the myocardium, FFAs released by EAT may have a direct effect on the myocytes and coronaries due to a complete lack of a fibrous fascial layer between the two. A large release of FFA may lead to cardiac lipotoxicity (Iacobellis et al., 2011).

Patients with increased EAT (measured on computed tomography; CT) have increased LV mass index (LVMI), large left atrial size (LA), and high E/e’ velocity by echocardiography (Butler et al., 2009; Brankovic et al., 2018; Acquarone et al., 2019). The association between EAT and LV parameters persist upon adjusting for obesity markers (BMI, waist circumference), and traditional CV risk factors (Kim et al., 2021). Epicardial AT may increase the myocardial fat content and interstitial fibrosis that likely affects myocardial contractility as evidenced by reduced global longitudinal strain (Ng et al., 2018). Elevated EAT also results in reduced peak VO^2^ consumption and peripheral extraction in patients with HfpEF, indicating a worse hemodynamic profile in these patients (Pugliese et al., 2021b).

Finally, in a recent analysis of MESA, EAT also was associated with an increased risk of incident HF (Kenchaiah et al., 2021). High EAT volumes defined as > 70 cm^3^ for women and > 120 cm^3^ for men correlated with a twofold increased incidence of HF in women and 53% higher risk in men. Increased EAT predominantly enhanced the risk of HFpEF (*p* < 0.001) and not HFrEF (*p*.31) (Table 2).

### Role of Chronic Kidney Disease in Heart Failure With Preserved Ejection Fraction

Nearly 50% of patients with HFpEF have chronic kidney disease (CKD) (Redfield et al., 2003; Yancy et al., 2006). The etiology of CKD is multifactorial in HFpEF (van de Wouw et al., 2019). Comorbidities and HF contribute to microvascular dysfunction that causes and perpetuates both renal dysfunction and LV remodeling (van de Wouw et al., 2019). Chronic kidney disease is associated with premature vascular aging (Laurent et al., 2006) leading to macrovascular and microvascular dysfunction. Advanced atherosclerosis and arteriosclerosis worsen HTN that increases LV workload and exacerbates LVH and LVDD (Borlaug and Kass, 2011). Arteriosclerosis also leads to pulsatility (Mitchell, 2008) in the coronary microvascular bed that promotes microvascular disruption and CMD (Safar et al., 2015; van de Wouw et al., 2019). At a molecular level, CKD worsens the above mentioned pro-inflammatory pathways leading to increased ROS production, reduced local NO availability, and CMD (Rosner et al., 2012; Paulus and Tschope, 2013).

**FIGURE 2:**
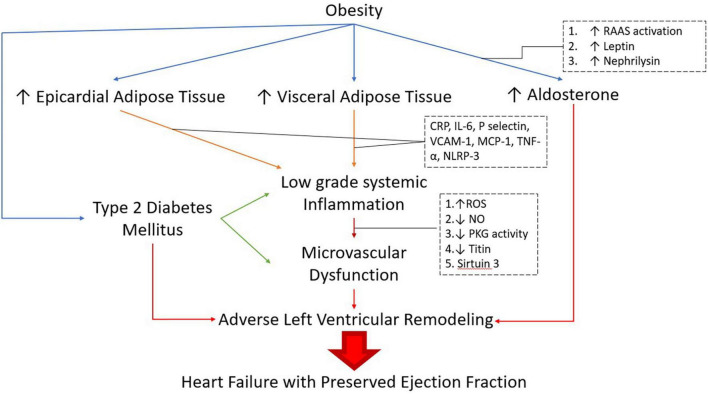
Pathogenesis of obese-DM-HFpEF. RAAS: Renin-Angiotensin-Aldosterone system, CRP: C-Reactive Protein, IL -6: lnterleukin-6, VCAM-1: Vascular cell adhesion molecule 1, MCP-1: monocycte chemoattractant protein-1,TNF-α: Tumor Necrosis Factor alpha, NLRP-3: Nod-Like receptor protein 3, ROS: Reactive oxygen species, NO: Nitric Oxide, PKG: Protein Kinase G.

Obesity itself leads to a glomerulopathy, i.e., obesity-related glomerulopathy (ORG) that is characterized by maladaptive glomerular hypertrophy and focal segmental glomerulosclerosis (D’Agati et al., 2016). Other pathways of obesity-related CKD involve alteration of adipokines (Briffa et al., 2013), activation of Renin-Angiotensin-Aldosterone System (RAAS) (Upadhya et al., 2020), and ectopic lipid accumulation within the kidneys (Escasany et al., 2019) (Figure 2).

Management of CKD in patients with obesity and HFpEF is challenging. Targeting the RAAS showed promise in retrospective studies with a greater reduction of proteinuria seen in obese than non-obese individuals (Praga et al., 2001; Mallamaci et al., 2011; Tsuboi et al., 2013). However, there is still resistance in initiating RAAS inhibitors due to the fear of downstream CKD progression and hyperkalemia.

Weight loss improves proteinuria and has a favorable effect on the estimated glomerular filtration rate (Saiki et al., 2005; Shen et al., 2010; Friedman et al., 2013). Bariatric surgery markedly reduces proteinuria (Fowler et al., 2009; Huan et al., 2009). However, bariatric surgery is associated with long-term renal complications like nephrolithiasis and oxalate nephropathy (Turgeon et al., 2012; Lieske et al., 2015).

#### Linking Diabetes Mellitus, Obesity, and Heart Failure With Preserved Ejection Fraction: A Clinical Perspective

Besides T2D macrovascular complications, the direct effects of T2D on the myocardium have received increased attention over the last decade (Jia et al., 2018).

At a molecular level, patients with T2D-HFpEF have increased t-tubule density and lower collagen deposition when compared with HFrEF. Patients with T2D-HFpEF also have impaired diastolic calcium homeostasis including reduced sarco/endoplasmic reticulum Ca^2+^-ATPase activity indicating a different pathophysiological process when compared to non-T2D HFpEF (Frisk et al., 2021).

Though BMI is a poor marker of VAT and EAT (Le Jemtel et al., 2018), elevated BMI indirectly suggests a high prevalence of both. MESA, BMI, and VAT was significantly elevated in patients with incident HFpEF (29.9 vs. 27.8, *p* 0.01; 230.7 cm^3^ vs. 162.6 cm^3^ *p* < 0.001, respectively) (Rao et al., 2018). Patients with EAT have elevated BMIs and increased VAT, further linking BMI as an indirect measure of VAT and EAT (Kenchaiah et al., 2021).

In HFpEF, patients with T2D commonly have higher BMIs than their non-diabetic counterparts. In an ancillary study of the Phosphodiesterase-5 inhibition to improve clinical status and Exercise capacity in Diastolic HF (RELAX) trial (Redfield et al., 2013; Reddy et al., 2019), BMI were 37.1 and 30.7 kg/m^2^ vs. 30.7 kg/m^2^ in patients with and without T2D. Unsurprisingly, patients with T2D-HFpEF had more severe initial presentations and more frequent hospitalizations. They also had more LVH and higher filling pressures (E/e’) by echocardiography. Cardiac Magnetic Resonance Imaging (CMR) reveals a trend toward higher LV Mass and higher levels of fibrosis in T2D than patients with non-T2D HFpEF (Lejeune et al., 2021). Patients with diabetes had high BMIs (31 vs. 27 kg/m^2^, *p* 0.001) and had an increased rate of mortality and hospitalization for HF (HR 1.72 95% C.I. 1.1–2.6, *p* 0.011).

A common feature of both T2D and HFpEF is exercise intolerance (EI) (Upadhya et al., 2015a; Pandey et al., 2021). The cause of exercise training (ET) is multifactorial (Pandey et al., 2021) in T2D and HFpEF with impairment in cardiac performance (Pugliese et al., 2021a) and skeletal muscle metabolism/perfusion (Espino-Gonzalez et al., 2021). Obesity and T2D significantly contribute to EI in HFpEF. Obesity-induced sarcopenia exacerbates muscle mass loss due to aging and worsens EI (Upadhya et al., 2015b). Type 2 diabetes lowers exercise capacity through impaired cardiac energetics (Levelt et al., 2016) and skeletal muscle oxygen extraction and metabolism (Nesti et al., 2020). Clinically, EI leads to poor quality of life (Salzano et al., 2021), frequent re-hospitalizations, and early mortality in T2D and HFpEF (Pugliese et al., 2020). Thus, reversal of EI is an important therapeutic target in patients with T2D-HFpEF. improves peak VO^2^ (Demopoulos et al., 1997) and quality of life in patients with systolic dysfunction (Fleg et al., 2015). In HFpEF, ET improves peak VO^2^ and quality of life independent of improvement in cardiac systolic or diastolic function (Pandey et al., 2015b). The effects of ET on skeletal muscle perfusion and metabolism warrant investigation in T2D-HFpEF.

Patients with T2D HFpEF have an increased risk of mortality (Yusuf et al., 2003; MacDonald et al., 2008; Massie et al., 2008). In the I-PRESERVE trial, patients with T2D also had a higher prevalence of coronary artery disease (CAD) and percutaneous coronary intervention/coronary artery bypass graft (PCI/CABG) indicating an increased risk of macrovascular disease (Massie et al., 2008). Of note, patients with and without T2D had similar LVEFs, and patients with T2D had significantly greater LV Mass and DD despite having a lower prevalence of hypertension (HTN) (Kristensen et al., 2017). The greater LV remodeling in patients with T2D was likely related to greater BMIs (31 + /6 vs. 29 ± 5 kg/m^2^, respectively). Moreover, in the T2D cohort, 52% of the patients had a BMI > 30 kg/m^2^ vs. 38% of the patients in the non-T2D cohort (Kristensen et al., 2017).

Lastly, the duration and severity of T2D in HFpEF affect outcomes. In a sub-analysis of the Treatment Of Preserved Cardiac function heart failure with an Aldosterone Antagonist (TOPCAT) trial (Pitt et al., 2014), patients from the Americas (*n* = 1765 patients) were analyzed into 3 subgroups, patients with T2D treated with insulin (ITDM, *n* = 390 patients), patients with T2D not on insulin (NITDM, *n* = 406 patients), and patients withoutT2D (*n* = 969 patients) (Huynh et al., 2019). The ITDM cohort had a longer duration of T2D and higher BMI when compared with NITDM and non-T2D patients. The ITDM cohort also had worse LVDD and increased LV Mass. Unsurprisingly, ITDM patients had the worst outcome profile with a 50% increase in all-cause and CV mortality that was elevated when compared to NITDM patients alone. The risk of adverse outcomes was similar in NITDM and non-T2D. Thus, obesity and T2D are additive risk factors in patients with T2D-HFpEF (Huynh et al., 2019). However, obesity directly impacts the severity of T2D as well as HFpEF. Obesity clearly worsens outcomes in T2D. Increasing insulin resistance leads to increased production of insulin from pancreatic β-cells that eventually cannot meet glycemic demands. The ectopic pancreatic fat deposition also contributes to β-cell dysfunction and thereby to T2D (Ishibashi et al., 2020). Thus, treatment of the obese HFpEF phenotype needs to target obesity and T2D.

## Therapeutic Advances for Obese-T2D-Heart Failure With Preserved Ejection Fraction Phenotype

### Targeting Coronary Microvascular Dysfunction

Therapy in HfpEF, specifically in the obese-T2D-HfpEF phenotype is searching for novel therapeutic approaches. Targeting CMD is an innovative approach but so far results have not been promising (Redfield et al., 2013, 2015; Borlaug et al., 2018). Increasing NO availability and enhancing cGMP have been disappointing. In multiple trials looking at phosphodiesterase inhibitors and oral nitrates, increasing NO has failed to improve quality of life or exercise capacity in HfpEF. Most trials recruited patients with high BMI, severe LV concentric remodeling, and advanced LVDD at baseline. Hence, extensive collagen deposition and LV stiffness may account for the neutral findings (Samson and Le Jemtel, 2021).

Given the neutral findings of the above trials, increasing NO may not be the most effective way to remedy endothelial dysfunction. Vericiguat, an sGC stimulator bypasses NO production and can stimulate the production of cGMP that as previously mentioned prevents further LV remodeling. Vericiguat did not improve the primary endpoints of NT-ProBNP levels and left atrial volumes but did improve quality of life in a clinical trial (Pieske et al., 2017). A lower BMI than in prior trials (∼30 kg/m^2^ in all groups) hints at a low prevalence of VAT and EAT in this population.

Regardless, despite the high prevalence of CMD in HFpEF (Shah et al., 2018), targeting CMD does not seem to be therapeutically fruitful.

### Targeting Mineralocorticoid Receptors

Sodium retention secondary to increased aldosterone production plays a major role in obese-HFpEF. It accounts for the responsiveness to diuretics but excess natriuresis can accelerate renal dysfunction (Gupta et al., 2012). Experimentally, MRAs reduce oxidative stress (Gorini et al., 2019), cardiac inflammation (Tesch and Young, 2017) and fibrosis (Borlaug and Kass, 2011), and improve diastolic LV filling pressures (Pandey et al., 2015a). Spironolactone improved LV filling pressures and exercise capacity in patients with HFpEF. In T2D, spironolactone improves insulin resistance (Olatunji et al., 2017) and albuminuria (Makhlough et al., 2014; Selvaraj et al., 2018). The effects on diabetic nephropathy are mixed with delayed progression in type 1 (Schjoedt et al., 2005) but not T2D (Tofte et al., 2020).

In the TOPCAT trial, patients with obesity and T2D benefited the most from spironolactone (Cohen et al., 2020). Maximum reduction of the primary endpoint (All-cause death and HF hospitalization) was noted in patients with a BMI > 33 kg/m^2^ (Elkholey et al., 2021). A similar benefit was seen in patients with high waist circumference (HWC) (Men > 102 cm and women > 88 cm) indicating that spironolactone was more beneficial in patients with increased VAT. The beneficial effect of spironolactone in obese and HWC patients is a reduction in HF hospitalization. Quantification of VAT may help better risk stratify patients who benefit from MRAs. The promising pre-clinical favorable metabolic effects of finerenone (Marzolla et al., 2020) suggest that MRAs may benefit adjunct obese-T2D-HFpEF phenotype.

### Targeting Obesity and Diabetes Mellitus

The pathophysiology of HFrEF highlights worsening LVEF due to the progression of eccentric LV remodeling which leads to symptom deterioration and eventual patient decline. The success of neurohormonal modulation in HFrEF is based on the ability of pharmacotherapy and device therapy ability to reserve LV eccentric remodeling. In contrast, neurohormonal modulation does not reverse LV concentric remodeling in HFpEF (Lam et al., 2018; Upadhya and Kitzman, 2020). Hence, the most effective therapies in HFrEF do not lower mortality in HFpEF (Massie et al., 2008; Pitt et al., 2014; Solomon et al., 2019). As previously mentioned, obesity, specifically VAT and EAT, drive LV remodeling (Yan et al., 2020). Obesity leads to T2D hence aggressive weight management will benefit patients with HFpEF and T2D.

Treating obesity is complex and involves lifestyle/behavioral modifications (LBM) as the first step, then pharmacotherapy and bariatric surgery as the next step. Newer advances in anti-diabetic medications have led to a successful strategy of targeting obesity and HF in patients with T2D changing the management paradigm for these patients.

#### Glucagon-Like Peptide-1 Analogs

Glucagon-like peptide-1 (GLP-1) analogs are coming back in T2D and recent trials demonstrate efficacy in CV disease (Verma et al., 2018) and weight loss (Wilding et al., 2021). GLP-1 receptors are expressed in various organs like the heart, kidney, and pancreas. GLP-1 agonists reduce ROS production by the endothelium and systemic inflammation. It may contribute to their beneficial effect on LV diastolic function studies (Nguyen et al., 2018; Bizino et al., 2019).

GLP-1 agonists have also been shown to be effective in reducing EAT which is a target in HFpEF (Dutour et al., 2016; Iacobellis et al., 2017). In 95 patients with T2D, liraglutide plus metformin was associated with a 36% reduction in EAT when compared with metformin alone (Iacobellis et al., 2017). In 44 patients, exenatide was also associated with a ∼10% reduction in EAT when compared to 1.2% in the standard of care arm (Dutour et al., 2016).

Reduction of adiposity is an essential therapeutic aim in obese-T2D-HFpEF. Before recent semaglutide trials, pharmaceutical agents approved for weight loss by the Food and Drug Administration (FDA) at best resulted in 7% weight loss (Srivastava and Apovian, 2018). GLP-I analogs have been shown to cause an average weight loss of 2.9 kg 95% C.I. 2.2–3.6 kg in 21 trials and 6,411 patients (Vilsboll et al., 2012). The finding of the recent Four Semaglutide Treatment Effect in People with Obesity (STEP 1–4) trials was more promising (Davies et al., 2021; Rubino et al., 2021; Wadden et al., 2021; Wilding et al., 2021). Subcutaneous semaglutide was compared with intensive LBM in successive steps in patients with and without T2D. Semaglutide reduced body weight by 10% in 75% of patients, 15% in 56% of patients, and 20% in 36% of patients. In contrast in a veteran’s affairs study (Maciejewski et al., 2016), patients with gastric bypass (GB) reduced weight by 27.5% (95% C.I. 23.8–31.2%), sleeve gastrectomy (SG) by 17.8% (95% C.I. 9.7–25.9%) underlining the magnitude of weight loss achieved by semaglutide. The cardiovascular and outcome effects of GLP-1 analogs need to be investigated in patients with obese-T2D-HFpEF.

#### Sodium-Glucose Co-transporter 2 Inhibitors

Sodium-glucose co-transporter 2 inhibitors (SGLT-2i) are extremely beneficial in HFrEF (McMurray et al., 2019; Packer et al., 2020). The actions are multiple (Lopaschuk and Verma, 2020) and include weight loss, increased diuresis, improved endothelial function, reduced inflammation, and cardiac remodeling prevention. Weight loss is modest (Mean 1.5–2 kg) (Liu et al., 2015; Maruthur et al., 2016; Zaccardi et al., 2016), and slightly greater in patients with T2D than those non-T2D (Pereira and Eriksson, 2019). Weight-loss lasts up to 4 years (Del Prato et al., 2015) and is dose-dependent (Cai et al., 2018). However, the weight loss induced by glycosuria leads to a compensatory increase in appetite and thereby caloric intake (Ferrannini et al., 2015). Thus, SGLT-2i must be combined with other medications for a lasting effect on weight (Leibel et al., 1995). SGLT-2i reduces perivascular AT thereby lowering leptin release and loco-regional inflammation (Iborra-Egea et al., 2019). In patients with T2D with CAD, SGLT2i reduces EAT, TNF-α, and plasminogen activator inhibitor-1 (Sato et al., 2018). SGLT2i effect on TNF-α leads to the improved endothelial secretion of NO and reduced CMD (Juni et al., 2019). Several experimental models have demonstrated the benefits of SGLT-2i on cardiac remodeling (Lambers Heerspink et al., 2013; Verma et al., 2016; Connelly et al., 2019). In a randomized clinical trial, patients treated with empagliflozin had a significantly lower LV mass index when compared with placebo at 6 months (Connelly et al., 2019). Reduced cardiac fibrosis and inhibition of the mammalian target of rapamycin pathway may alleviate LV remodeling (Lee et al., 2019; Kang et al., 2020).

Recently, in the empagliflozin in HFpEF (EMPEROR-PRESERVED) trial (Anker et al., 2021), empagliflozin did reduce the combined endpoint of CV death or hospitalization but did not reduce significantly reduce CV death alone. The results were underwhelming for patients with typical HFpEF as the benefits were mostly noted in patients with LVEF < 50% compared to LVEF > 60% i.e., HF mid-range-EF. Of note, patients with BMI > 30 kg/m^2^ (HR 0.85 C.I 0.7–1.03) did not derive as much benefit as those with BMI < 30 kg/m^2^ (HR.7 C.I 0.62–0.88). The findings of the awaited dapagliflozin trials (Solomon et al., 2021) may strengthen EMPEROR-HF (Packer et al., 2020).

#### Role of Metabolic Surgery

Metabolic surgery, specifically GB, is the most effective intervention for weight loss. It prevents the occurrence of HFpEF in patients with severe obesity. However metabolic surgery has complications and requires careful and long-term monitoring. Compared with LBM, metabolic surgery results in a greater weight loss and a 23% risk reduction in HF (Sundstrom et al., 2017). Exercise-induced weight loss, an integral part of LBM, reduces EAT and thereby, the incidence of HF (Kim et al., 2009).

Weight loss has not been so far a therapeutic target in the management of HFpEF. Of note, regular aerobic exercise training is an arduous undertaking for severely or morbidly obese patients. Weight loss improved LV mass index (LVMI) in MESA with every 5% weight loss being associated with a 1.3% decrease in LVMI and LV mass-to volume ratio (*p* < 0.0001) measured by cardiac MRI (Shah et al., 2015). The Utah obesity study examined patients undergoing metabolic surgery and compared them with control patients with morbid obesity who did not undergo surgery. All patients underwent 2D echocardiography, and close monitoring (Adams et al., 2005). Mean baseline BMI was 48 and 44 kg/m^2^ in metabolic surgery and control patients. At 2 years, BMI was 32 and 44 kg/m^2^ in metabolic surgery and control patients. Patients who underwent metabolic surgery had significant reductions in LVMI and increases in right ventricular (RV) fractional area change at 2 years (Owan et al., 2011). Smaller studies reported similar findings (Karason et al., 1997; Ippisch et al., 2008; Aggarwal et al., 2016). The effects of caloric restriction and/or exercise were reported in older patients with obesity and HFpEF. After 20 weeks, body weight decreased by 4 kg (3%) in the exercise group, 7 kg (7%) in the caloric restriction group, and 11 kg (10%) in the combined group while it increased by 1 kg (1%) in the control group. Both caloric restriction and exercise independently improved exercise capacity [as measured by peak oxygen consumption (VO^2^)] and the effects of caloric restriction and exercise were additive. However, there was no difference in the quality of life (as reported on the Minnesota Living with Heart Failure Questionnaire) or LVMI in either group. High intensity and moderate continuous exercise regimens do not significantly improve in peak VO^2^ compared with guideline-directed exercise regimens (Mueller et al., 2021) (Table 3).

**TABLE 3:** Major studies addressing role of bariatric surgery in heart failure.

Bariatric surgery						
**Study name**	**Study type**	**Treatment modality**	**N**		**Median follow up**	**Main outcomes**
			**GB**	**LBM**		
Sundstrom et al. (2017)	Nationwide Registry	GB vs. LBM	25,804	13,701	4.1 years	• Patients undergoing GB lost 18.8 kg more weight at year 1 and 22.6 kg more weight at year 2 • HR for incident HF was 0.54 (C.I. 0.36–0.82) in GB patients • 10 kg weight loss was associated with a 23% reduction in incidence of HF (HR 0.77 C.I. 0.6–0.97) in both arms
Utah obesity study (Adams et al., 2005)	Prospective Cohort Study	GB vs. LBM	423	733	2 years	• Patients undergoing GB had marked weight loss (reduction in BMI with GB 15 kg/m^2^ vs. 0.03 kg/m^2^ in LBM) • The GBS group had reductions in LV mass index and RV cavity area • GBS group also had increased LV midwall fractional shortening and RV fractional area change

*GB, Gastric Bypass; LBM, Lifestyle and behavioral modifications; HR, Hazard ratio; HF, Heart Failure; LV, Left ventricle; RV, Right Ventricle.*

Adherence to LBM is strongly recommended for patients with obesity, T2D, and HFpEF and physicians need to be pro-active to effectively help patients lose weight. Metabolic surgery though beneficial is marred by strict indications (Ayinapudi et al., 2020) and multiple complications (Ma and Madura, 2015; Surve et al., 2018). Randomized controlled trials of metabolic surgery and LVM are clearly needed in patients with severe and morbid obesity with HFpEF.

## Conclusion

Heart failure with preserved ejection fraction remains a therapeutic conundrum. The obese-T2D phenotype has distinct pathophysiology encompassing inflammation, CMD, and LV remodeling. Obesity is at the crux of the pathophysiology and weight reduction must be prioritized in these patients. Quantification of VAT and EAT may better help risk-stratify patients at greatest risk of HFpEF and further studies are needed to assess their impact on management. Mineralocorticoid receptor antagonists and anti-diabetic agents like semaglutide and SGLT-2 inhibitors hold promise as useful adjunct agents for obese-T2D-HFpEF and should be studied in randomized clinical trials. Lifestyle and behavioral modifications should be offered to all patients and metabolic surgery may be considered in patients with BMI > 35 kg/m^2^.

## Author Contributions

AD-P and TL contributed to conception and design of the article, wrote the manuscript, and drafted the figures. TT and RS edited certain sections of the manuscript. All authors contributed to manuscript revision, read, and approved the submitted version.

## Conflict of Interest

The authors declare that the research was conducted in the absence of any commercial or financial relationships that could be construed as a potential conflict of interest.

## Publisher’s Note

All claims expressed in this article are solely those of the authors and do not necessarily represent those of their affiliated organizations, or those of the publisher, the editors and the reviewers. Any product that may be evaluated in this article, or claim that may be made by its manufacturer, is not guaranteed or endorsed by the publisher.
